# Patient information leaflets for Transrectal Ultrasound guided prostate biopsy: Results of North Thames deanery survey

**DOI:** 10.1186/1756-0500-3-27

**Published:** 2010-01-28

**Authors:** Iqbal Shergill, Kishore Bahl, Muhammad Farjad, Claire Phipps, George Fowlis

**Affiliations:** 1 Department of Urology, North Middlesex University Hospital NHS Trust, London, UK

## Abstract

**Background:**

We evaluated the quality of patient information leaflets for Trans-Rectal Ultrasound guided prostate biopsies (TRUS-Bx) in North Thames region. TRUS-Bx information leaflets were requested from 24 hospitals in the region. All hospitals were contacted by telephone, and non-responders were followed-up by postal survey. Leaflets received were evaluated for a clear description of the procedure, directions to TRUS-Bx location, a clear description of the procedure, contact for queries/concerns, information about preparation prior to procedure, information about regular medication, information on how to obtain results, instructions for follow-up arrangements, analgesia used and risk of morbidity/mortality. Additionally, the leaflets were evaluated for diagrams to clarify the procedure and the anatomy, and sources of additional information, such as reference to published articles or prostate cancer patient support groups/internet websites.

**Findings:**

In summary, a total of 17 leaflets (77%) were received. Of these, the majority (94%) had a clear description of the procedure, contact for queries/concerns (82%), information about preparation prior to TRUS-Bx (71%). Directions to TRUS-Bx location (29%), and analgesia used (35%), was very poorly described, and information on obtaining results and follow-up arrangements were described in only 12 (71%) leaflets. Complications such as risks of infection, haematuria, haematospermia and rectal bleeding, were generally explained (71%-76% of leaflets), urinary retention was mentioned in only 5 (29%) leaflets and mortality in only 1 case. Descriptive diagrams of the procedure and prostate anatomy were very rarely used, and sources of additional information were limited to 1 published article and reference to 1 prostate cancer support group.

**Conclusions:**

This study demonstrates that there is large variation in the information supplied in TRUS-Bx patient information leaflets in the North Thames region, with some leaflets lacking vital information. It is proposed that a standard patient information leaflet incorporating all the factors in the checklist should be designed, with the incorporation of a new BAUS procedure specific consent form for TRUS-Bx.

## Findings

### Introduction

A recent UK white paper suggests that all patients should have improved access to high quality information, health professionals should communicate more effectively with patients, and there should be a nationally coordinated process to produce and deliver information [1]. Patient information leaflets are a good example of how this can be achieved, for several important reasons [2]. Importantly, they allow improved communication between doctors and patients, which is especially useful in the process of informed consent, as well as reducing patient anxiety [3].

Trans-Rectal Ultrasound guided prostate biopsy (TRUS-Bx) is a common outpatient procedure that is undertaken when a diagnosis of prostate cancer is suspected. Although it is relatively quick to perform and is done under local anaesthetic, it is nevertheless an invasive procedure, that may cause significant anxiety to patients. In addition, it may potentially result in morbidity and mortality. Complications include septicaemia, haematuria, haematospermia, rectal bleeding and urinary retention. Despite national advice that the patient should be provided with comprehensive information about TRUS-Bx procedure [4,5], the short contact time between a patient and the doctor in many urology diagnostic units may be insufficient to fully inform and prepare the patient adequately for the procedure. This is especially the case with the introduction of the one-stop clinic, for suspected prostate cancer via the two-week-wait referral system, where patients are seen, assessed and then undergo TRUS-Bx within a matter of minutes. Interestingly, even though several written information sheets for informed consent exist for urological procedures [6] TRUS-Bx is not included. Furthermore, there is no national standardised patient information sheet that exists, contrary to suggestions in the recent UK white paper, for diagnostic procedures such as TRUS-Bx.

Hence, with the understanding that a lack of appropriate health information can have potentially serious or even fatal consequences [7], we evaluated the quality of patient information leaflets for TRUS-Bx, in the North Thames Deanery.

### Materials and Methods

TRUS-Bx information leaflets were requested from the 24 hospitals in the North Thames region that perform this procedure. All hospitals were initially contacted by telephone, and non-responders were then followed-up by postal survey. Leaflets received were then evaluated against a checklist of 17 items (Table 1). In the absence of an established checklist for this purpose, several sources were used to derive this checklist. This included The British Society of Gastroenterology [8], guidelines for items that should be included in any information sheet, originally developed for patients undergoing upper gastrointestinal endoscopic procedures, a similar study in the Urology literature investigating flexible cystoscopy information leaflets [9] as well as the Prostate Cancer Risk Management Programme (PCRMP) guidance on undertaking a TRUS-Bx of the prostate [4] and the NICE guidelines for prostate cancer [5]. Additionally, the leaflets were evaluated for diagrams to clarify the procedure and the anatomy, and sources of additional information, such as reference to published articles or prostate cancer patient support groups/internet websites.

**Table 1:** Summary of checklist and results from the audit.

Checklist Item	(n)	%
Directions to the location of TRUS-Bx	5	29%

A clear description of the procedure	16	94%

A contact number for queries or concerns before or after the procedure	14	82%

Information about preparation prior to procedure	12	71%

Information about regular medication	12	71%

Information on how to obtain the result of the TRUS-Bx	11	65%

Instructions for follow-up arrangements	10	59%

Analgesia mentioned?	6	35%

Risk of infection	12	71%

Risk of haematuria	13	76%

Risk of haemospermia	12	71%

Risk of rectal bleeding	13	76%

Risk of retention of urine	5	29%

Mortality	1	6%

Diagrams to clarify the procedure	3	18%

Diagrams to clarify the anatomy	3	18%

Sources of additional information	2	12%

## Results

The results are summarized in Table 1. In summary, a total of 17 leaflets (77%) were received. Of these, the majority (94%) had a clear description of the procedure, contact for queries/concerns (82%), information about preparation prior to TRUS-Bx (71%). Directions to TRUS-Bx location (29%), and analgesia used (35%), was very poorly described, and information on obtaining results and follow-up arrangements were described in only 12 (71%) leaflets. Complications such as risks of infection, haematuria, haematospermia and rectal bleeding, were generally explained (71%-76% of leaflets), urinary retention was mentioned in only 5 (29%) leaflets and mortality in only 1 case. Descriptive diagrams of the procedure and prostate anatomy were very rarely used, and sources of additional information were limited to 1 published article and reference to 1 prostate cancer support group. The majority of leaflets were produced between 2003 and 2006 (n = 15). The date of production of the remaining 2, were not listed.

## Discussion

### Summary of findings

The current study demonstrates that there is large variation in the information supplied in these in the North Thames region, with some leaflets lacking vital information. This has the potential risk of causing uncertainty and misunderstanding for patients undergoing such a common outpatient procedure.

### Strengths and Weaknesses

One of the concerning findings from this survey is that the complication of urinary retention and death following TRUS-Bx of prostate was rarely described. PCRMP guidance clearly states that the patient should be provided with comprehensive information about the procedure, including estimates of the risks of potential complications and post-procedure events - this includes retention of urine and death [4]. Other complications were explained, but not in all of the leaflets. This suggests that important complications are being omitted which is contrary to national advice from PCRMP. An alternative explanation is that patients are being counselled thoroughly on the day of biopsy and are given information on discharge following the biopsy, as per PCRMP advice [4], although this cannot be inferred directly from this survey.

Information regarding preparation prior to procedure included requirements for antibiotic prophylaxis, which were variable, and the use of enemas, even though there is no requirement for patients to be given an enema before undergoing the procedure, according to PCRMP guidance [4].

Several leaflets provided no information about fasting in preparation for the procedure. This lack of information may have resulted in unnecessary discomfort to those patients who fasted, especially patients with diabetes. In addition, information and advice regarding regular medication was missing in almost a third of cases, and one leaflet stated "Do not stop taking your medication until advised". Clearly, this may have important implications regarding complications as anticoagulant therapy in the form of warfarin and anti-platelet agent clopidogrel which should be stopped before undertaking TRUS-BX [4,5]. According to our survey, although several leaflets stated that warfarin and clopidogrel should be stopped prior to TRUS-Bx, only one leaflet stated that bleeding was more likely if patients were taking warfarin. One leaflet stated "please inform us if you have a latex allergy", but this may reflect an increased prevalence of this problem in that hospitals catchment area.

Furthermore, only two leaflets gave advice on continuing the usual dose of any regular medication, apart from anticoagulants. Again, this lack of information is worrying, as it is possible that some patients may omit their drugs, such as those for diabetes, with dangerous consequences. One method of overcoming these problems is to involve patients in the development of patient information leaflets, in close conjunction with clinicians [2].

We were surprised to find that the type of analgesia to be used was only mentioned in a third of leaflets, especially as there is strong recent evidence suggest that it TRUS-Bx may be a painful procedure and that patient tolerance and comfort can be improved by analgesia [10].

Of all the leaflets, illustrations or diagrams were only used in six and only one used photographs. All illustrations, except one, was labelled, in accordance with the NHS Toolkit, which has been introduced by the Department of Health for help in producing patient information and endorses the use of illustrations and diagrams advising that they should be clearly labelled and not overwritten [11].

### Implications for practice and research

Although a significant number of leaflets had information regarding contact details (Urology department or Uro-Oncology Nurse Specialists numbers) only one leaflet made it clear which source of information was used to compile the publication, and only one leaflet provided any details of additional sources of support and information (a support association). These items are questions in the DISCERN tool [12,13], which was developed to assess a broad range of aspects of the quality of information. Although not used in this survey, as it specifically applies to treatment choices, DISCERN scores of relevant questions from the leaflets studied would have been very low, indicating low or moderate quality information. Furthermore, the PCRMP was not referenced in any of the leaflets, possibly as the majority of leaflets were old and had not been updated appropriately [13].

The implications, based on the information from this survey, are that a national standardised patient information sheet for TRUS-BX should be produced and a detailed audit, with laid-out criteria/standards, should be undertaken after a new leaflet has been designed.

In summary, results from this survey suggest that there is large variation in the information supplied in TRUS-Bx patient information leaflets in the North Thames region, with some leaflets lacking vital information. It is proposed that a standard patient information leaflet incorporating all the factors in the checklist should be designed, and in addition, we believe that templates from the NHS toolkit [11] as well as incorporation of a new BAUS procedure specific consent form would help standardise the quality and presentation of patient information for TRUS-Bx.

## Competing interests

The authors declare that they have no competing interests.

## Authors' contributions

IS conceived the study, participated in its design and coordination, helped collect the data and helped to draft the manuscript. KB participated in its design and coordination, helped collect the data and helped to draft the manuscript. MF participated in its design and coordination and collected the data.CP participated in its design and coordination and collected the data. GF participated in its design and coordination and helped to draft the manuscript and revised it critically for important intellectual content. All authors read and approved the final manuscript.
